# Combined exposure to silica nanoparticles and high-fat diet modulates metabolism-associated fatty liver disease via the gut–liver axis

**DOI:** 10.3389/fmicb.2026.1810542

**Published:** 2026-05-08

**Authors:** XueYan Zhang, Kaifeng Chen, Yifan Liang, Shuo Sun, Junjie Chen, Qian Wang, Fenghong Wang, Lei Zhang

**Affiliations:** 1 School of Public Health, Shandong Medical and Pharmaceutical University, Yantai, Shandong, China; 2 School of Public Health, North China University of Science and Technology, Tangshan, Hebei, China; 3 School of Public Health, Inner Mongolia Medical University, Hohhot, Inner Mongolia, China

**Keywords:** gut–liver axis, high-fat diet, metabolism-associated fatty liver disease, multi-omics, silica nanoparticles

## Abstract

**Background:**

The increasing prevalence of metabolism-associated fatty liver disease (MAFLD) is associated with environmental pollutants and dietary factors, yet the synergistic effect and underlying mechanism of silica nanoparticles (SiNP) and a high-fat diet (HFD) remain unclear.

**Methods:**

This study aimed to investigate the role of the gut–liver axis in MAFLD pathogenesis induced by co-exposure to SiNP and HFD, utilizing a multi-omics approach.

**Results:**

In this study, we found that combined SiNP and HFD exposure exacerbated liver injury, as evidenced by significant steatosis, inflammatory infiltration, fibrosis, and elevated serum ALT/AST levels. It impaired intestinal barrier integrity and induced gut microbiota dysbiosis, characterized by altered microbial richness and differential abundance of specific bacteria. Liver metabolomics revealed significant perturbations, with the riboflavin metabolism pathway being the most notably enriched. Key metabolites in this pathway, flavin mononucleotide (FMN) and flavin adenine dinucleotide (FAD) showed dose-dependent alterations. Correlation analysis underscored a strong link between specific gut microbes and riboflavin metabolism intermediates. Network toxicology identified six hub targets—IL-6, IL-1β, Casp3, Pparγ, Alb, and Tgfβ1 within the riboflavin metabolism network, and molecular docking confirmed their strong binding affinities with FMN and FAD.

**Conclusion:**

These findings demonstrated that combined exposure to SiNP and HFD induced gut–liver axis dysfunction and exacerbated MAFLD progression, which was mainly attributed to gut microbiota dysbiosis-mediated disruption of riboflavin metabolism. Our study highlighted the gut microbiota-riboflavin axis as a potentially promising intervention strategy for MAFLD, and identifies possible targets for the prevention and treatment of MAFLD.

## 1 Introduction

Metabolism-associated fatty liver disease (MAFLD) is a chronic hepatic condition strongly linked to metabolic abnormalities, characterized pathologically by excessive accumulation, inflammatory infiltration, and fibrotic progression (Ji et al., 2023). This disease exhibits significant associations with metabolic syndromes including obesity, insulin resistance, and type II diabetes mellitus. The global prevalence of MAFLD has risen dramatically in recent years, paralleling the worldwide adoption of Westernized dietary patterns and increasingly sedentary lifestyles, making it now the most prevalent chronic liver disease affecting human populations (Tian et al., 2022). Epidemiological data indicate MAFLD affects approximately 25% of the global population and 31.5% of Chinese adults, with prevalence rates continuing to escalate annually (Pipitone et al., 2023). Without effective intervention, MAFLD can further progress to non-alcoholic steatohepatitis, cirrhosis and even hepatocellular carcinoma, which is a serious threat to human health (De Cól et al., 2024). Although obesity is recognized as a major driver of MAFLD pathogenesis, emerging evidence suggests that widespread exposure to environmental pollutants (e.g., nanoparticles) may exacerbate the disease progression through poorly understood synergistic mechanisms (Li et al., 2024).

Silica nanoparticles (SiNP) are widely used in biomedicine, food additives and industrial materials due to their high specific surface area, excellent chemical stability and facile functionalization properties. However, the potential health risks of environmental and occupational exposure are of increasing concern. Studies have shown that SiNP can enter the body through inhalation, ingestion or dermal contact, and cross the biological barrier to accumulate in the liver and other organs, inducing oxidative stress, inflammatory reactions and metabolic disorders (Huang et al., 2022). Animal studies have revealed that long-term exposure to SiNP may disrupt hepatic lipid metabolism and promote steatosis, suggesting that SiNP could represent a novel environmental risk factor for MAFLD (Duan et al., 2018). It is worth noting that a high-fat diet (HFD), as a classic trigger of MAFLD, may have a synergistic effect with SiNP through lipotoxicity, insulin resistance and other pathways (Balasubramaniam et al., 2019), but the regulatory mechanism of MAFLD by combined exposure of the two has not been systematically studied.

Emerging evidence has demonstrated that intestinal dysbiosis plays a pivotal role in the development of MAFLD through the gut–liver axis. This microbial imbalance compromises intestinal barrier integrity, resulting in endotoxin translocation and pro-inflammatory cytokine release. These mediators subsequently activate the hepatic innate immune system through portal circulation, exacerbating hepatic lipid accumulation and fibrotic progression (Rao et al., 2021). Furthermore, gut microbiota-derived metabolites, particularly short-chain fatty acids and bile acids, have been shown to directly regulate host lipid metabolism and inflammatory signaling pathways (Martin-Gallausiaux et al., 2021). However, there are no studies on the role of SiNP and HFD in the development of MAFLD by interfering with the structure and function of the gut microbiota.

To address this research gap, we integrated metagenomics and metabolomics methods to explore the mechanism by which SiNP combined with HFD induce MAFLD in rats via the gut–liver axis. Furthermore, network toxicology and molecular docking techniques were applied to identify key targets for gut microbiota-derived metabolites in MAFLD. This study will provide a novel insight into the early prevention and intervention strategies for MAFLD from the perspective of the coordinated pathogenic effects of environmental pollutants and dietary factors.

## 2 Materials and methods

### 2.1 Reagents and materials

The standard feeds (TP2330055AC, Fat: 10% of total calories, Protein: 15% of total calories, Carbohydrate: 75% of total calories) and high-fat feeds (D12079B, Fat: 41% of total calories, Protein: 17% of total calories, Carbohydrate: 43% of total calories, 0.15% of cholesterol) were purchased from Jiangsu Nantong Trofeo Feed Science and Technology Co, Ltd., with the high-fat feeds stored at 4 °C. The SiNP (250 mg/10 mL suspension, particle size 100 nm) used in the experiment was obtained from Tianjin Bexler Chromatography Technology Development Centre. Transmission electron microscopy (TEM) was employed to analyze the particle size distribution, morphology, and degree of agglomeration of the SiNP. The physicochemical characterization of SiNP Hydrated particle size and zeta potential in different aqueous solutions were determined using dynamic light scattering (DLS).

### 2.2 Animal model

The animal experimental protocol for this experiment was approved by the Animal Care and Use Committee of Inner Mongolia Medical University (YKD202402114). Male Wistar rats (6–8 weeks old, 200 ± 20 g) were obtained from Beijing SPF Biotechnology Co., Ltd. In this study, male rats were selected for this study to eliminate the potential confounding effects of the female estrous cycle on metabolic phenotypes, to ensure the stability of the MAFLD disease model, and to adhere to the 3R principle by reducing individual variability, thereby maintaining data comparability with prior research. After 1 week of feeding, the rats were randomly divided into four groups (*n* = 9/group): control, HFD, SiNP exposure, and co-exposure of SiNP and HFD groups. The rats were exposed to SiNP by intratracheal instillation (10 mg/kg) every 4 days for 90 days and body weights were measured weekly throughout the experimental period. To ensure consistency, all intratracheal instillations of SiNP were performed at a fixed time of day, specifically between 8:00 p.m. and 10:00 p.m.

### 2.3 Sample collection

After 3 months of exposure, the rats were anaesthetized and approximately 4 mL of blood was collected via the abdominal aorta. Rats were anesthetized by intraperitoneal injection with either 4% chloral hydrate solution (400 mg/kg). The depth of anesthesia was confirmed by the absence of the toe-pinch and corneal reflexes prior to surgery. The rats were then euthanized, and liver, intestinal tissues, and intestinal contents were rapidly harvested. Finally, the liver coefficient was calculated [(liver weight/fasting body weight) × 100%]. Fresh tissue was excised and immediately cut into small pieces (approximately 50–100 mg). The samples were then wrapped in placed into cryovials, promptly snap-frozen in liquid nitrogen, and subsequently transferred to a −80 °C freezer for long-term storage.

### 2.4 Histopathological analysis

Four rats were randomly selected from each group for histopathological analysis. After paraffin-embedded sections of liver and intestinal tissues, hematoxylin–eosin (H&E) staining was performed to assess histomorphological features (Shamai et al., 2022). The process involves the sequential deparaffinization and rehydration of liver and intestinal tissue samples, followed by Hematoxylin and Eosin (H&E) staining, dehydration through a graded ethanol series, and mounting with a coverslip. The prepared slides are then examined under a microscope for histopathological evaluation. Masson trichrome staining to quantify collagen fiber deposition (Mao et al., 2016) to characterize the degree of fibrosis, respectively. Liver and intestinal tissues were deparaffinized, rehydrated, and mordanted in potassium dichromate overnight. Sections were then stained with iron hematoxylin, treated with Ponceau-acid fuchsin, differentiated in phosphomolybdic acid, and counterstained with aniline blue. After dehydration and mounting, the slides were examined microscopically.

### 2.5 Determination of serum and liver function indicators

Serum ALT (C009-2-1) and AST (C010-2-1) levels were measured using the microplate method according to the kit instructions (Nanjing Jiancheng Biological Engineering Institute). ALT and AST were considered the most specific and sensitive indicators for liver injury, while the AST/ALT ratio helped assess the severity and potential etiology of liver damage.

### 2.6 Macro-genome sequencing

Metagenomic sequencing was performed using intestinal content samples (200 mg per rat) that were collected and stored at −80 °C. Total microbial DNA was extracted with the QIAamp PowerFecal Pro DNA Kit (Qiagen, Germany) following the manufacturer’s protocol, and DNA quality was assessed by NanoDrop 2000 (OD260/280 = 1.8–2.0) and 1% agarose gel electrophoresis (main band >10 kb). Libraries were prepared using the Nextera DNA Flex Library Prep Kit (Illumina, USA), and paired-end sequencing (PE150) was conducted on the Illumina NovaSeq 6,000 platform with a target output of ≥20 Gb per sample. Host-derived reads were removed by aligning to the rat reference genome (Rnor_6.0) using Bowtie2 (v2.4.4), followed by downstream bioinformatics analyses. Raw sequencing data were deposited in the NCBI database, and assembly accuracy was validated by PCR–Sanger sequencing of randomly selected 5% of assembled contigs.

### 2.7 Liver metabolomics

50 mg of thawed rat liver tissue was homogenized in pre-chilled methanol/water (4:1, v/v), sonicated on ice for 10 min, and centrifuged (12,000 rpm, 4 °C, 15 min). The supernatant was collected, filtered through a 0.22 μm membrane, and stored at −80 °C until analysis; pooled quality control (QC) samples were included to monitor instrument stability. Metabolomic profiling was performed by ultra performance liquid chromatography-quadrupole-time of flight mass spectrometry system (UHPLC-Q-TOF/MS, Agilent 1290/6545, USA), using water with 0.1% formic acid (A) and acetonitrile with 0.1% formic acid (B) as the mobile phases. The methanol (LOT 832115) and acetonitrile (LOT 962758) used in the experiment were purchased from RiMedCo Chemical Company, USA. The internal standard, 2-chloro-L-phenylalanine (C804740-1 g), was purchased from Shanghai Macklin Biochemical Technology Co., Ltd. Chromatographic conditions: ACQUITY UPLC HSS T3 column (2.1 mm × 100 mm, 1.8 μm); mobile phase A was 0.1% formic acid aqueous solution, and B was acetonitrile; gradient elution program: 0–2 min 5% B, 2–10 min 5–95% B, and 10–12 min 95% B, with a flow rate of 0.3 mL/min. Mass spectrometry conditions: scan range of 50–2000 m/z; scan time of 0.2 s, scanning range m/z 50–1,000; capillary voltage of 2.0 kV; low collision energy of 6.00 eV, high collision energy ramp of 20 eV to 75 eV; source temperature of 120 °C, desolvation temperature of 500 °C; cone gas flow of 50 L/h, desolvation gas flow of 1,000 L/h. Raw data were processed in Progenesis QI for peak picking, alignment, and total ion normalization, and metabolites were annotated using Metascope against an in-house library and public databases (HMDB and METLIN) based on retention time, accurate mass, MS/MS spectra, isotopes, and collision cross-section, retaining features with confidence scores >30. Principal Component Analysis (PCA) and Partial Least Squares Discriminant Analysis (PLS-DA) were used to identify differential metabolites (VIP > 1.0, Student’s *t*-test *p* < 0.05), and pathway enrichment was performed using KEGG via MetaboAnalyst 5.0 (*p* < 0.05).

### 2.8 Network toxicology and molecular docking analysis

Network toxicology and molecular docking were integrated to identify potential mechanisms linking riboflavin metabolism to SiNP/HFD-induced MAFLD. Two key riboflavin-related metabolites (FMN and FAD) were selected, and their candidate targets were retrieved from ChEMBL^1^ and the Comparative Toxicogenomics Database (CTD)^2^ (Zhang et al., 2025). MAFLD-associated genes were collected from GeneCards^3^ using the keywords “liver injury,” “MAFLD,” and “liver fibrosis,” and overlapping targets were identified using Venn analysis. These intersecting targets were imported into STRING and Cytoscape to construct protein–protein interaction (PPI) networks. Molecular docking was performed using protein structures from AlphaFold, binding evaluation via Prodigy, and visualization in PyMOL. Functional enrichment (GO and KEGG) was conducted using database for annotation, visualization and integrated discovery (DAVID),^4^ with significance defined as *p* < 0.05.

### 2.9 Statistical analysis

All data are presented as mean ± SD. Normality and homogeneity of variance were assessed using the Shapiro–Wilk and Levene’s tests, respectively. Group comparisons were performed using Student’s *t*-test or the Mann–Whitney *U* test, as appropriate. Spearman correlation analysis combined with Sankey diagrams was used to visualize multi-omics associations among key gut microbiota, hepatic metabolic alterations, and riboflavin metabolism. Statistical analyses were conducted in SPSS 26.0 and GraphPad Prism 8.3, with two-tailed *p* < 0.05 considered significant.

## 3 Result

### 3.1 Characterization of SiNP

The TEM and DLS showed that favorable dispersion characteristics (Wang et al., 2022) with a near-spherical morphology, demonstrating ≥99.9% purity and a specific surface area of 60 m^2^/g, average particle size is 62.13 nm. Furthermore, the hydrated particle size and zeta potential of SiNP in deionized water, saline, and DEME were relatively stable, which reflected the good stability and dispersion of SiNP in diverse solution (Supplementary Figure S1).

### 3.2 Combined exposure to SiNP and HFD causes liver injury

**Figure 1:**
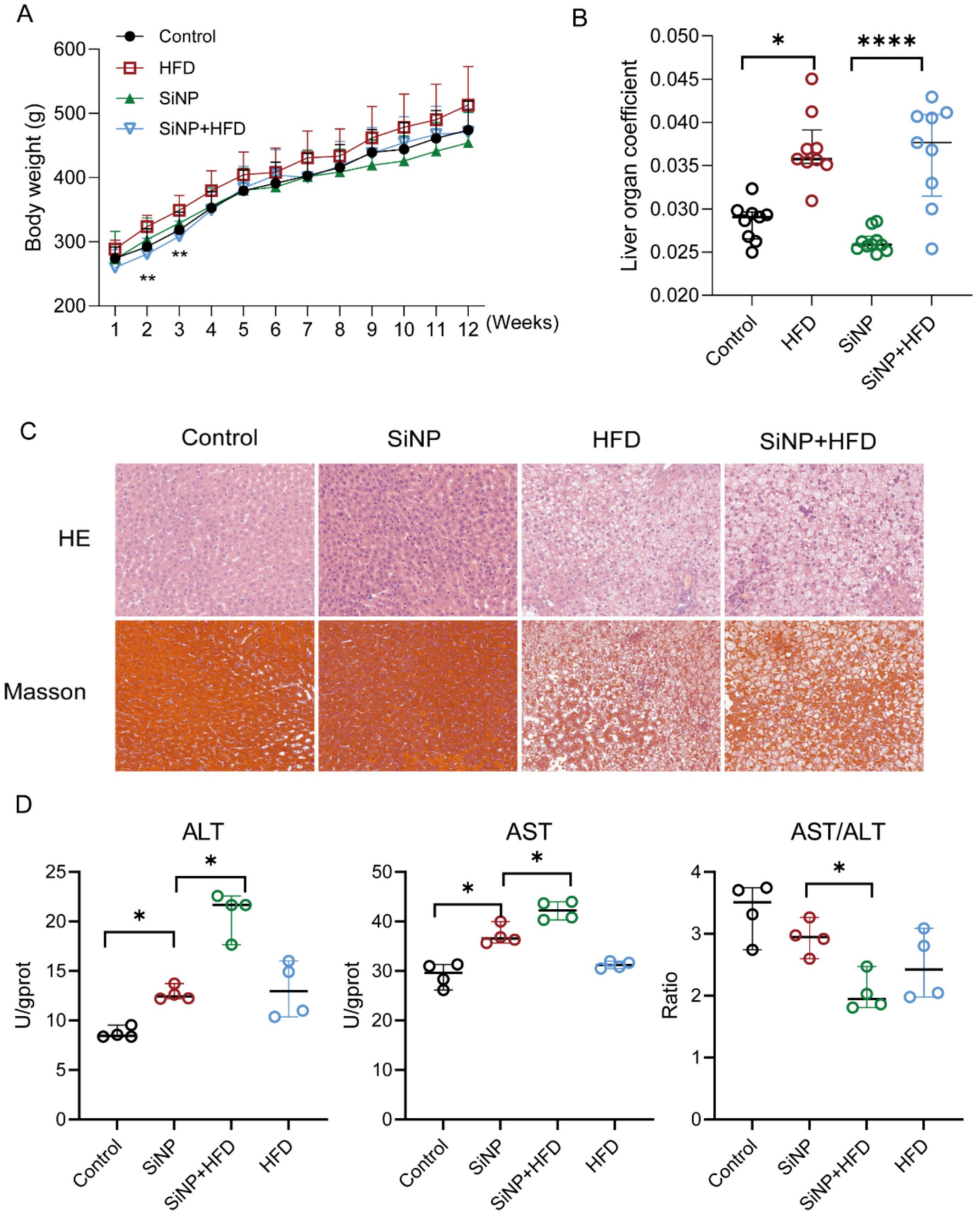
Effects of combined SiNP and HFD exposure on rats’ liver. **(A)** Changes in body weight of rats after combined exposure to SiNP and HFD (*n* = 9/group); **(B)** Changes in liver-body ratio of rats after combined exposure to SiNP and HFD (*n* = 9/group); **(C)** Morphologic changes in liver histopathology revealed by H&E and Masson staining (*n* = 4/group); **(D)** Serum and liver function biochemical indicators tests (*n* = 4/group). ^*^p < 0.05; ^**^p < 0.01.

Overall toxicity from combined SiNP and HFD exposure was evaluated by weekly body weight monitoring and the liver/body weight ratio. As exposure progressed, body weights diverged among groups (Figure 1A). The liver coefficient differed significantly between the SiNP group and the SiNP + HFD co-exposure group (Figure 1B).

H&E staining showed the SiNP group exhibited mild hepatic steatosis and focal inflammatory cell infiltration, whereas that of the SiNP and HFD co-exposure group showed disorganized hepatocyte arrangement, a large number of intracytoplasmic lipid droplet vacuoles, extensive ballooning, and periportal lymphocytic infiltration. Masson staining further revealed the progressive changes in collagen deposition. In the SiNP group, collagen fibers were focally aggregated around the hepatic sinusoids; whereas the SiNP and HFD co-exposure group exhibited dense bridging fibrosis connecting multiple portal areas to central veins (Figure 1C). Further, the results showed that serum ALT and AST levels in rats were significantly elevated in the SiNP-exposed versus SiNP and HFD co-exposure group, while AST/ALT was consistently down-regulated (Figure 1D). Taken together, these results indicated that the combined exposure of SiNP and HFD led to liver injury in rats.

### 3.3 Combined exposure to SiNP and HFD causes impairment of intestinal barrier

In the control group, intestinal tissue showed intact mucosal structure with well-organized crypts and villi and minimal inflammatory cell infiltration. H&E staining and AB-PAS staining revealed that rats in the HFD group showed mild shortening of villi and crypt hyperplasia, along with increased lymphocyte and neutrophil infiltration in the lamina propria. The SiNP group showed similar structural alterations, suggesting disruption of the intestinal epithelial barrier. Notably, The SiNP and HFD co-exposure group showed increased intestinal histopathologic changes, with widened gaps in the colonic mucosal epithelium, and atrophy of glandular cells in the lamina propria, including severe villous atrophy, crypt distortion, and inflammatory cell aggregation, suggesting a synergistic effect of SiNP and HFD on intestinal injury (Supplementary Figure S2).

### 3.4 Combined exposure to SiNP and HFD leads to alterations in gut microbiota abundance and function

**Figure 2:**
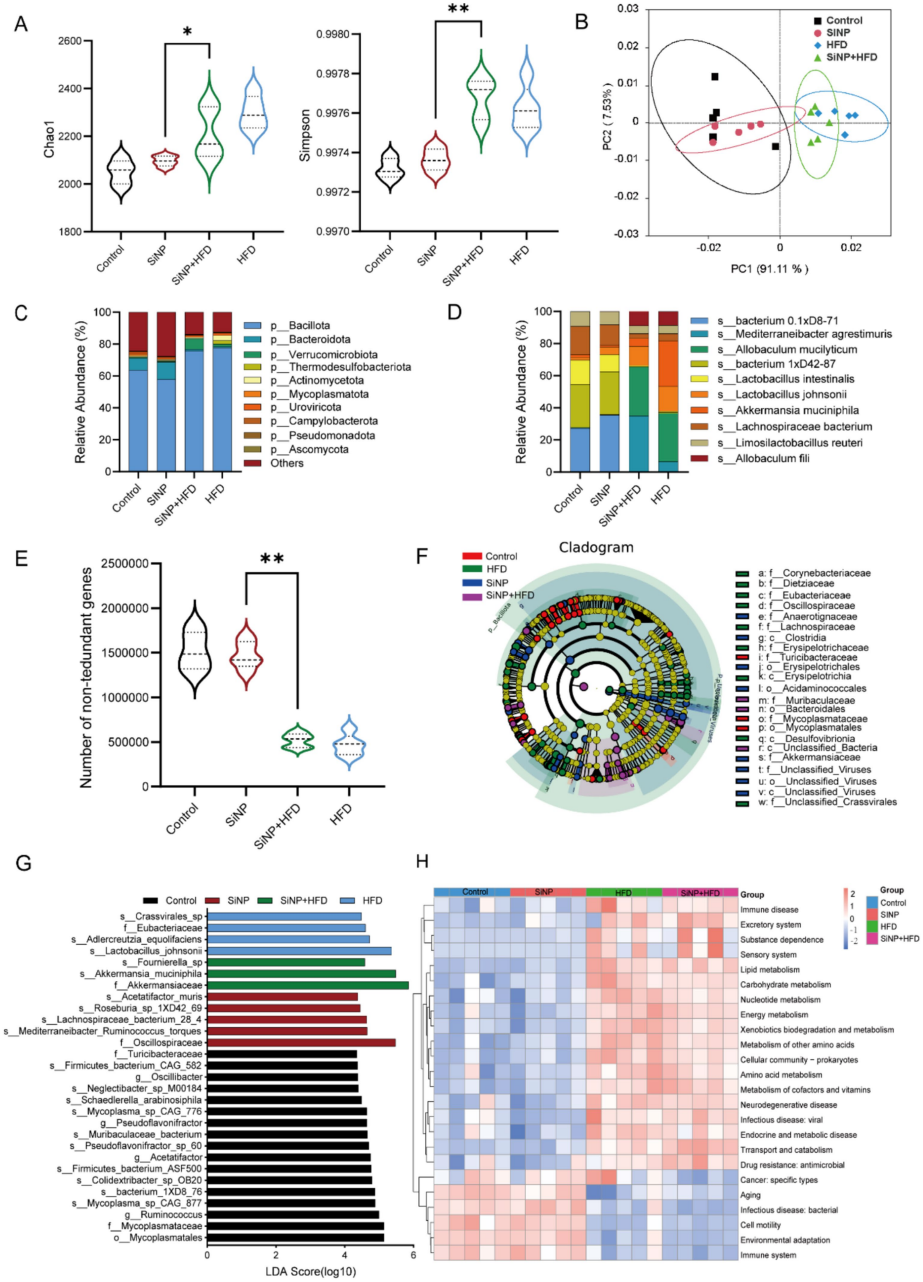
Macrogenomic analysis of rat intestinal contents (*n* = 5/group). **(A)** Alpha diversity measured by Chao1 and Simpson of patients with the four groups; **(B)** Principal coordinate analysis (PCoA) Bray-Curtis distance shows that microbial compositions were different between the four groups. **(C)** Stacked taxonomic bar plots at the phylum level stratified; **(D)** Stacked taxonomic bar plot at the species level stratifie; **(E)** Number of non-redundant genes detected between the four groups **(F)** Phylogenetic tree show differentially abundant microbial species analyzed by linear discriminant analysis (LDA) effect size (LEfSe) analysis (log_10_[LDA score] > 4); **(G)** LEfSe analysis defines significantly differentially abundant microbial species in the four groups (log_10_[LDA score] > 4); **(H)** Heat map of functional enrichment of four groups of differential microorganisms.

Metagenomic results indicated that the Chao1 and Simpson indicators both were significantly higher in the SiNP and HFD co-exposure group than in the SiNP group, indicating higher species richness and better evenness (Figure 2A). Furthermore, the Principal Coordinate Analysis (PcoA) based on Bray-Curtis distances indicated significantly different gut microbial clusters between the four groups (*p* < 0.05) (Figure 2B). At the phylum level, p_Bacillota was the predominant bacterial phylum in rat, with relative abundances of four groups were 63.71, 57.91, 75.68, 77.79%. Moreover, the abundance in the SiNP and HFD co-exposure group was significantly higher than in the Control and SiNP groups (*p* < 0.05) (Figure 2C). At the species level, the SiNP and HFD co-exposure group exhibited a significantly higher relative abundance of s_editerraneibacter agrestimuris than the other groups (Figure 2D).

The number of non-redundant genes detected across the four groups was significantly higher in the SiNP and HFD co-exposure group compared to the SiNP group (Figure 2E). Furthermore, Linear discriminant analysis Effect Size (LefSe) identified 29 taxa with significantly different abundances (Supplementary Figure S3). Under the criterion of Linear Discriminant Analysis (LDA) > 4, 17, 5, 3, and 4 taxa were enriched in the Control, SiNP, SiNP + HFD, and HFD groups, respectively (Figures 2F,G). Notably, s_Akkermansiaceae had a high LDA score in the SiNP + HFD group, suggesting it as a key biomarker and indicating that co-exposure may promote its expansion. Functional annotation of the differential microbes primarily involved lipid, carbohydrate, nucleotide, and energy metabolism, as well as metabolism of cofactors and vitamins, leading us to focus next on hepatic metabolic alterations (Figure 2H).

### 3.5 Liver metabolomic alterations due to combined exposure to SiNP and HFD

**Figure 3:**
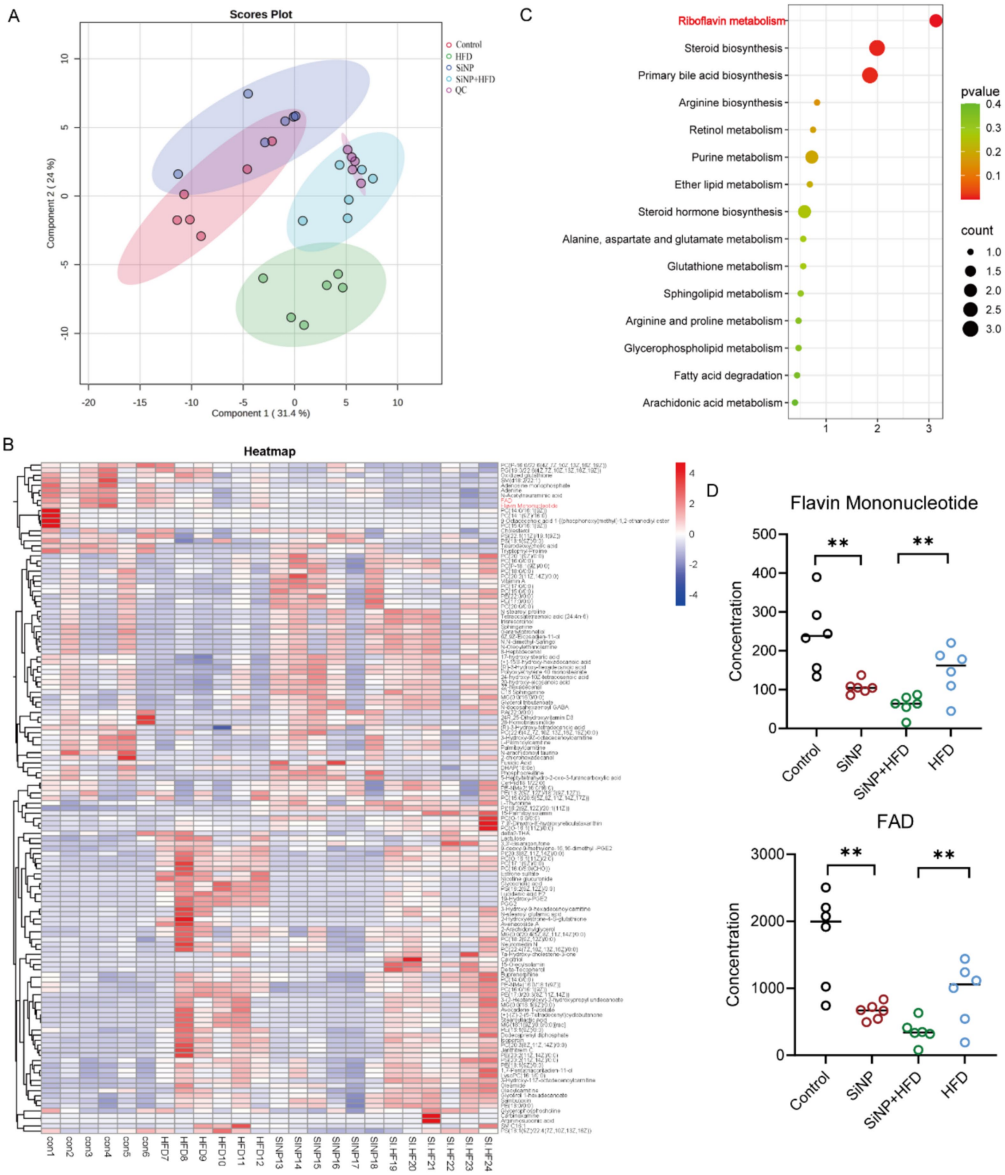
Alterations in the liver metabolome of the rats (*n* = 6/group). **(A)** PLS-DA results showed that distinct features of the liver metabolome between the four groups. **(B)** Heatmap depicting metabolites with significantly different abundances between the four groups. **(C)** Biological pathways enriched by differential liver metabolites between the four groups. **(D)** Bar graph showing significant differences in liver metabolite abundance between the four groups (Wilcoxon test).

Based on the metabolic pathways identified from the metagenomic results, metabolomic analysis was performed to alterations in liver metabolites. PLS-DA analysis revealed distinct clustering among groups, with particularly pronounced separation between the SiNP and SiNP and HFD co-exposure group, indicating significant alterations in endogenous metabolites within rat liver tissue after exposure (Figure 3A). At FDR-adjusted *p* < 0.05, we identified 133 significantly altered metabolites. Pathway enrichment highlighted riboflavin metabolism, arginine and proline metabolism, and bile acid biosynthesis (Figure 3B).

Pathway analysis in MetaboAnalyst 5.0 (*p* < 0.05, impact > 0.2) confirmed that SiNP and HFD primarily perturbed riboflavin metabolism, arginine and proline metabolism, and bile acid biosynthesis (Figure 3C). Within riboflavin metabolism, the key metabolites riboflavin mononucleotide (FMN) and FAD changed in a dose-dependent manner (Figure 3D). Collectively, these results suggested that gut microbiota could contribute to SiNP + HFD induced MAFLD by modulating the riboflavin metabolic pathway.

### 3.6 Combined macro-genomic and metabolomic analysis

**Figure 4:**
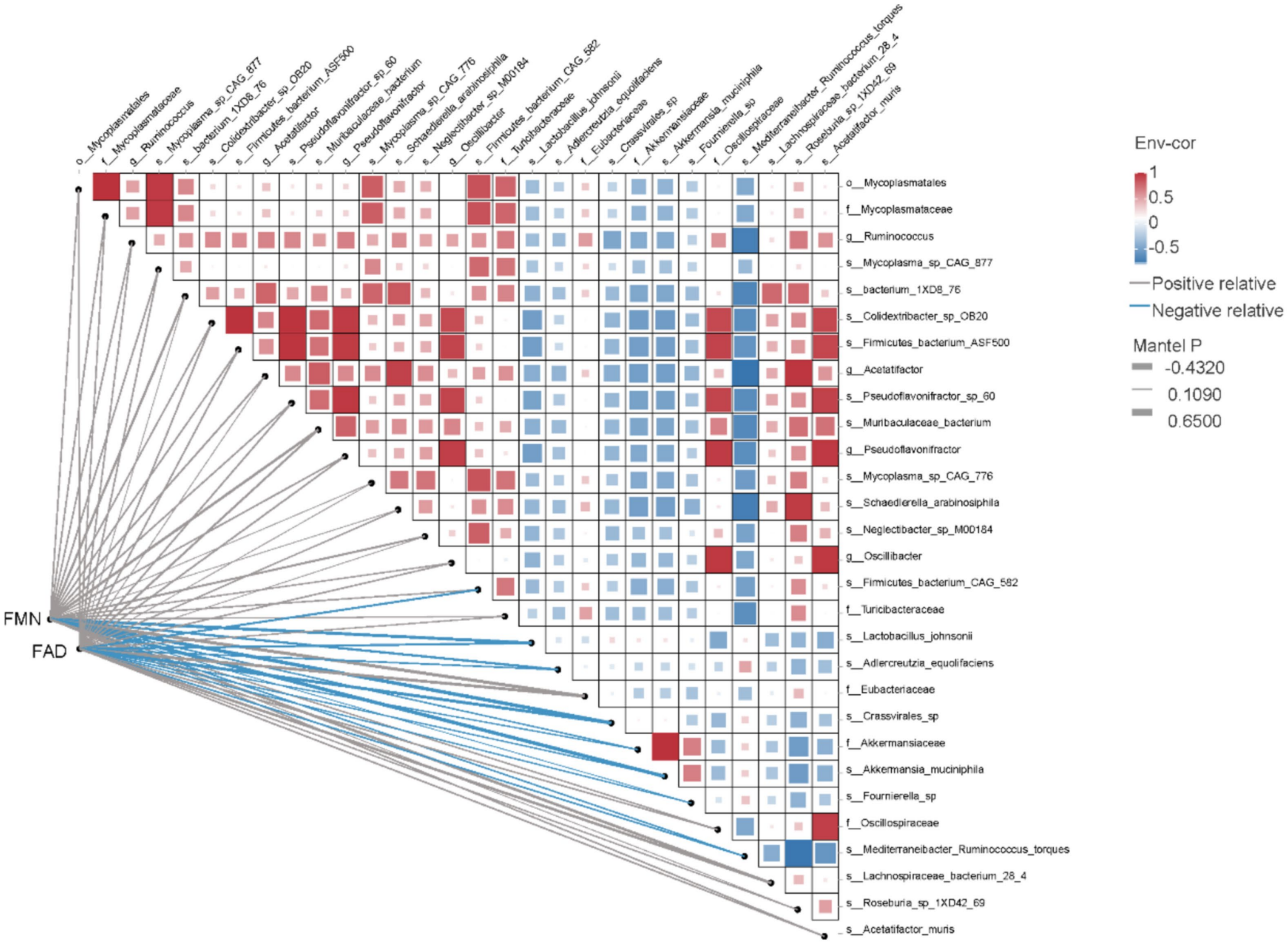
Dynamic network heatmap of FMN and FAD with 29 diffential bacterial species.

In this dynamic network heat map, we explored the relationship between Riboflavin metabolism and gut microbiome by Pearson correlation and mantel test. Significant associations (*p* < 0.05) existed in FMN for 14 bacterial metabolic microorganisms, of which those correlation coefficient greater than 0.6 were o__Mycoplasmatales, f__Mycoplasmataceae, s__Mycoplasma_sp_CAG_877, s_Firmicutes_bacterium_GAG_582, all were positively correlated with FMN. Significant associations (*p* < 0.05) existed in FAD for 13 bacterial metabolic microorganisms, of which those greater than 0.6 were o__Mycoplasmatales, s__Mycoplasma_sp_CAG_877, g_Ruminococcus, s_Colidextribacter_sp_OB20, s_pseudoflavonifractor_sp_60, g_Pseudoflavonifractor and s_Acetatifactor_muris. It is positively correlated with FAD (Figure 4).

### 3.7 Screening of potential targets in MAFLD induced by SiNP and HFD

**Figure 5:**
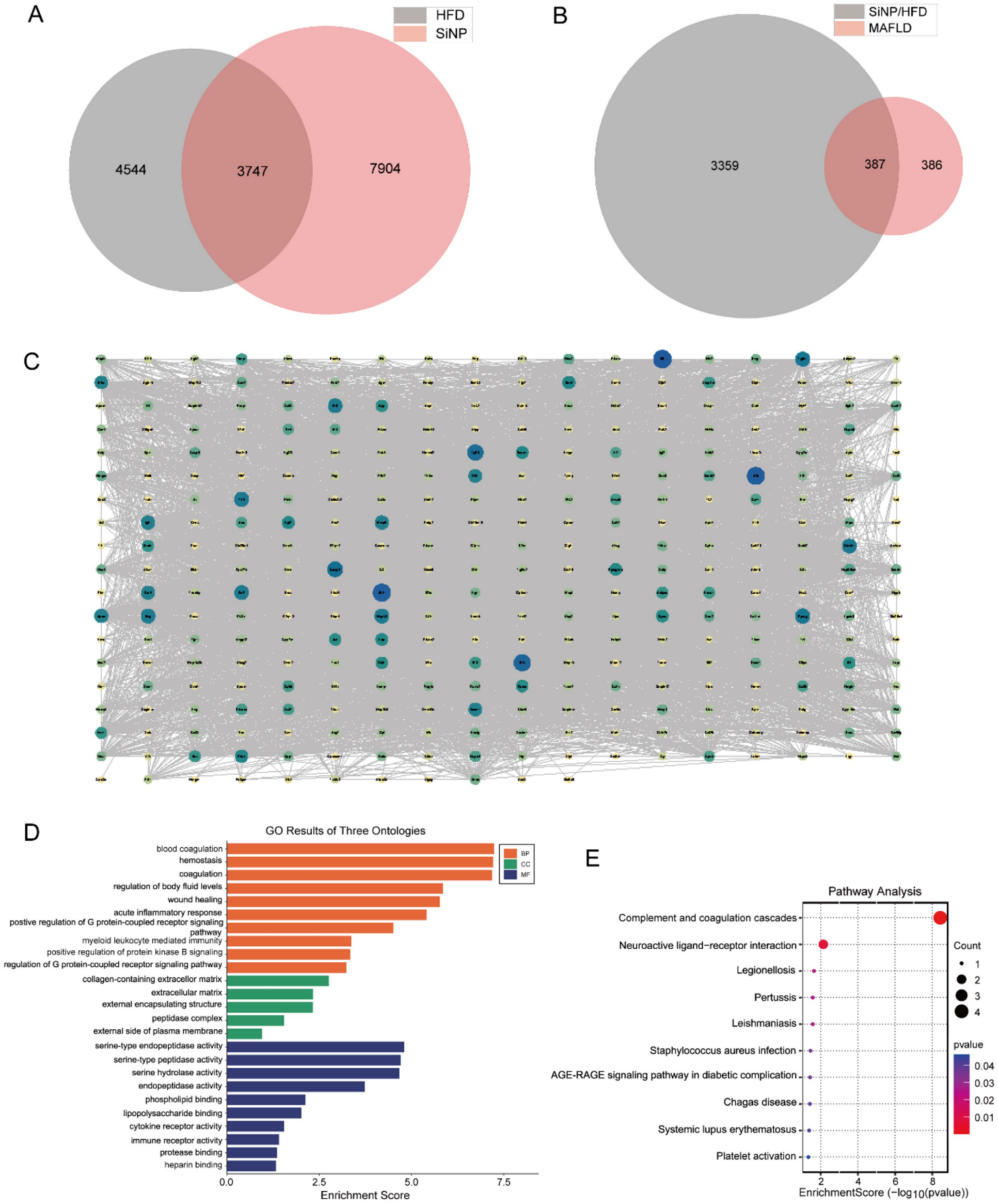
Predict the potential targets and mechanisms through network toxicology. **(A)** The Venn diagram shows the predicted number of targets associated with 18 FMN and 31 FAD. **(B-E)** PPI network of overlapping targets constructed via topological algorithms. (G&E) Bar graph of GO and KEGG enrichment analysis of overlapping targets.

To explore how SiNP and HFD contribute to MAFLD, we used a network toxicology framework to assess their potential target overlap with MAFLD-related genes. We first identified 11,651 SiNP-associated targets and 8,291 HFD-associated targets from the ChEMBL database, and retrieved 773 MAFLD-related targets from GeneCards. Venn analysis showed 387 shared targets among SiNP, HFD, and MAFLD (Figures 5A,B). To delineate potential mechanisms, we constructed a PPI network and performed functional enrichment analyses (Figure 5C).

GO enrichment indicated that the overlapping targets were mainly involved in blood coagulation, hemostasis, coagulation, and acute inflammatory responses. Enrichment of key targets further highlighted collagen-containing extracellular matrix, extracellular matrix, and external encapsulating structures, with molecular functions dominated by serine-type endopeptidase activity, serine-type peptidase activity, and serine hydrolase activity (Figure 5D). KEGG analysis showed significant enrichment in complement and coagulation cascades, neuroactive ligand-receptor interaction, and legionellosis (Figure 5E).

### 3.8 Identification of core targets in MAFLD induced by SiNP and HFD

**Figure 6:**
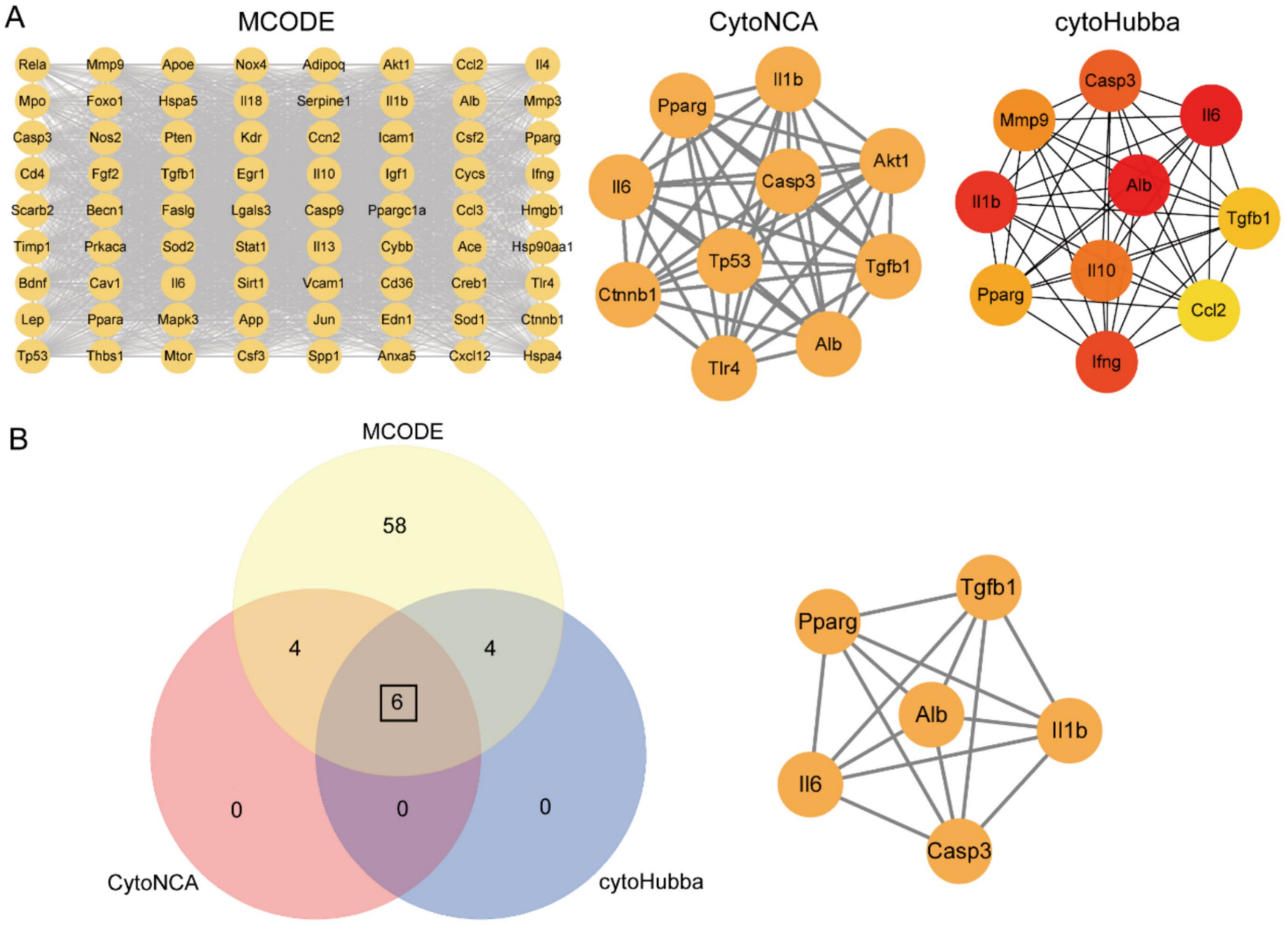
Predict the hub targets and mechanisms of riboflavin through network toxicology. **(A)** Using Cytosccape software to identify subnetworks using the MCODE, CytoNCA, and cytoHubba plugins. **(B)** Intersection of targets identified by MCODE, CytoNCA, and cytoHubba plugins.

To further explore the core network within the PPI, we utilized the subnetwork with the highest MCODE score, comprising 72 nodes and 2014 edges. Repeatedly obtain centrality scores using the CytoNCA plugin to identify 10 key nodes. And using the cytoHubba plugin to obtain a ranked list of key nodes and subnetworks, 10 key nodes were identified (Figure 6A). By taking the intersection of key nodes identified by the MCODE, CytoNCA, and Hubba plugins, we obtained six hub genes (IL-6, IL-1β, Casp3, Pparγ, Alb and Tgfβ1) (Figure 6B). Notably, all six hub genes were present in the highest-scoring subnetwork identified by MCODE, further underscoring their significance in MAFLD induced by SiNP and HFD.

### 3.9 Molecular docking analysis of riboflavin metabolism and hub targets

**Figure 7:**
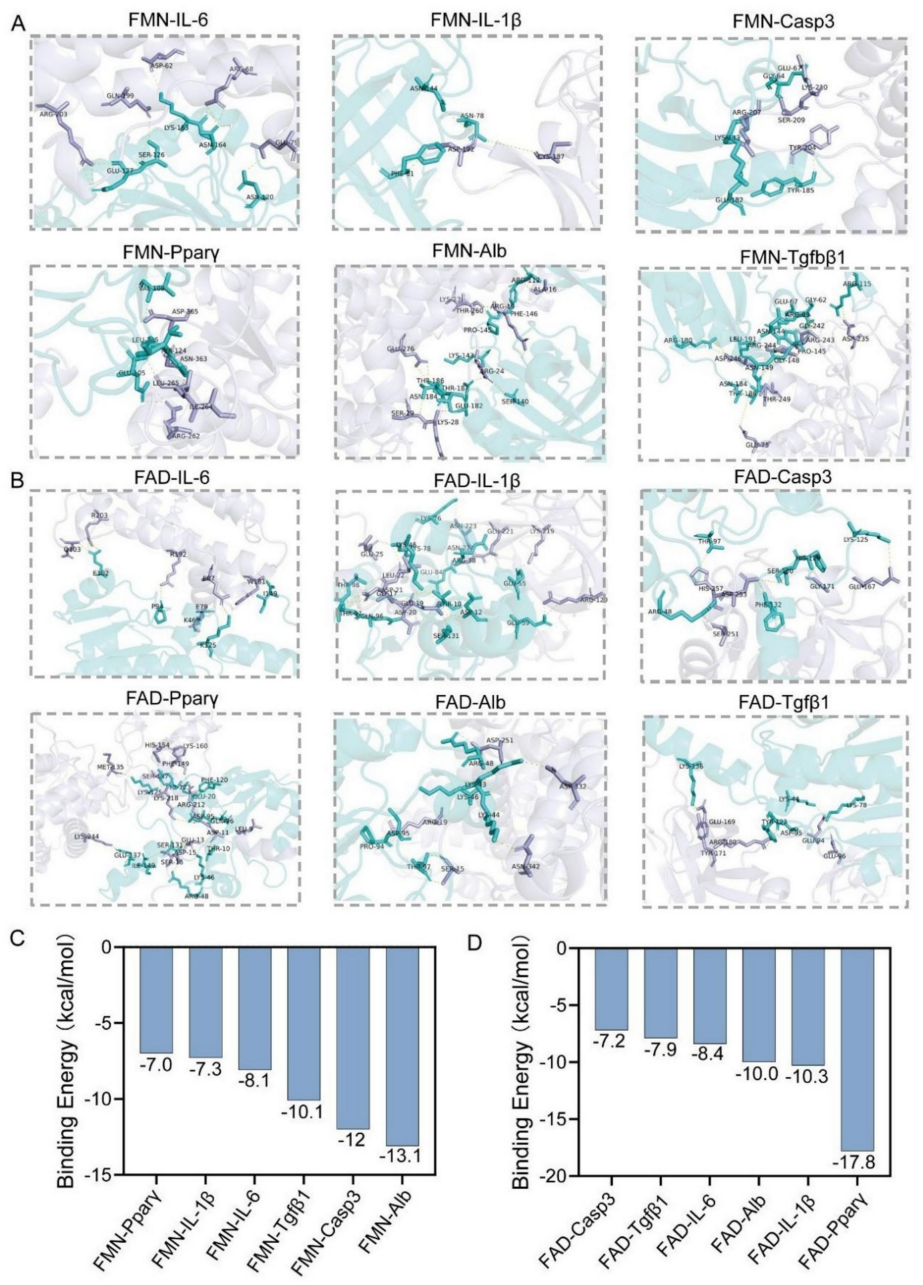
Three-dimensional structure of the receptor and ligand shown by molecular docking. **(A)** FMN molecular docking with six core targets. **(B)** FMN molecular docking with six core targets. (**C** and **D**) The binding energies of six hub targets.

By molecular docking, the interactions between FMN and FAD and their six core genes (IL-6, IL-1β, Casp3, Pparγ, Alb and Tgfβ1) were investigated (Figures 7A,B). Molecular docking results show that FMN has the strongest binding energy with Alb, while FAD has the strongest binding energy with Pparγ (Figures 7C,D). This result highlighted their potential role in the molecular mechanism of riboflavin-induced MAFLD.

## 4 Discussion

This study systematically investigated the role of the gut–liver axis in MAFLD following combined exposure to SiNP and HFD using metagenomics and metabolomics approaches. We found that combined SiNP and HFD exposure disrupted the intestinal barrier and gut microbiota in rats, with riboflavin metabolism emerging as a key pathway in MAFLD induction. Furthermore, network toxicology and molecular docking techniques revealed six key targets through which riboflavin metabolism induces MAFLD. This study provides novel evidence linking MAFLD to environmental pollution and unhealthy lifestyles, offering a foundation for precision prevention and treatment strategies.

This study aims to establish a long-term low-dose SiNP respiratory exposure model. Combining the occupational exposure limit for silica dust in workplace air (5 mg/m^3^, GBZ 2.1–2019) and physiological parameters of adults under light physical labor conditions with the deposition efficiency of approximately 60 nm-diameter nano-SiO₂ particles in human lungs, we estimate the exposure dose for an adult during a single workday to be approximately 0.37 mg/kg. Referencing the human-to-rats dose conversion factor provided by the FDA, this dose was correspondingly converted to an exposure dose for rats, yielding approximately 10.34 mg/kg. Based on the above calculations, we determined 10 mg/kg as the chronic poisoning concentration for SiNP in this study.

Emerging evidence highlights the synergistic detrimental effects of combined exposure to SiNP and HFD on liver health. SiNP, ubiquitous in environmental and industrial settings, have been shown to disrupt hepatic lipid metabolism and induce hepatotoxicity, though their role in MAFLD progression remains understudied (Abulikemu et al., 2023; Zhu et al., 2022). Respiratory or oral exposure to SiNP exacerbates HFD-induced liver steatosis, inflammation, and fibrosis, as demonstrated in rodent models, where SiNP altered the liver proteome and promoted microvesicular steatosis (Abulikemu et al., 2023; Stachyra et al., 2023). The gut–liver axis serves as a critical mediator in this interplay; HFD-induced gut dysbiosis impairs intestinal barrier function, elevates endotoxemia, and activates pro-inflammatory pathways in the liver, while SiNP further disrupt gut microbiota homeostasis and bile acid metabolism (Chu et al., 2025; Stachyra et al., 2023; Xu et al., 2021; You et al., 2025). For instance, SiNP orally administered to HFD-fed mice exacerbated lipid metabolism disorders in the gut–liver axis, with proteomic analyses revealing downregulation of metabolic pathways (Li et al., 2025; Sun et al., 2021). Conversely, interventions targeting the gut–liver axis (such as probiotics, dietary nanoparticles like ginger-derived GDNP, or plant-based compounds) have shown promise in mitigating HFD- and SiNP-induced liver damage by restoring microbial equilibrium, reducing systemic inflammation, and improving lipid homeostasis (Kumar et al., 2022; Wang et al., 2024; Xue et al., 2021). These findings underscore the need to evaluate combined environmental and dietary stressors in MAFLD pathogenesis, emphasizing the gut–liver axis as a therapeutic target.

A study has shown that Bacillota and cause obesity-related diseases, inflammatory bowel disease, and colorectal cancer risk (Andoh, 2016). In the fecal macrogenomic analysis of rats in this study, Bacillota at the gate level was significantly higher in the SiNP and SiNP and HFD co-exposure group than in the control group, suggesting that Bacillota has a role in intestinal injury and MAFLD under the combined exposure of SiNP and HFD, which is consistent with the findings. The bacterium 0.1xD8-71 has not been studied in detail at this time (Huang et al., 2024). Macrogenomics facilitated the study by accurately analyzing rat gut species composition and screening for differential microbes. Colonies of Fournierella have been shown in animal studies to cause metabolic disorders in mice induced by HFD. Reduced levels of *Akkermansia muciniphila* and Akkermansiaceae are thought to be associated with the lower incidence of disease. Most of these are metabolic disorders and inflammatory diseases, including obesity and type 2 diabetes (Zhang et al., 2019). In this study, microbial communities comprising Fournierella, *Akkermansia muciniphila* and Akkermansiaceae were significantly enriched in the group co-exposed to SiNP and HFD, suggesting these differential microbes might play a crucial role in the promotion of MAFLD induced by co-exposed to SiNP and HFD.

Riboflavin, also known as Vitamin B₂, was a water-soluble vitamin belonging to the B-complex vitamin family. It played a critical role in human energy metabolism, cellular growth, and antioxidant defense mechanisms. As a component of the coenzymes FAD and FMN, it participated in the oxidative metabolism of carbohydrates, fats, and proteins, facilitating energy extraction (Suwannasom et al., 2020). Riboflavin regulates energy metabolism by activating primary metabolic pathways and is involved in energy balance homeostasis (da Silva-Araújo et al., 2025). The role of riboflavin has also been dealt in the prevention of a wide array of health diseases like migraine, anemia, cancer, hyperglycemia, hypertension, diabetes mellitus, and oxidative stress directly or indirectly (Thakur et al., 2017). However, there is virtually no research on riboflavin and MAFLD. In this study, screening of differential metabolites and identification of their primary enriched pathways revealed that the gut microbiome modulates SiNP and HFD concomitant exposure-induced MAFLD through riboflavin metabolism. These results demonstrated strong correlation with prior studies. Here, we systematically integrated metabolomic analyses to reveal the details of the response of rats hepatocytes to the exposure of SiNP and HFD. This study contributes to expanding our understanding of riboflavin’s role in health and facilitates its application in toxicological assessments.

Previous studies have highlighted riboflavin’s regulatory role in hepatic lipid metabolism. Riboflavin supplementation has been shown to suppress oxidative stress and lipogenesis, thereby improving hepatic steatosis and injury in mutant models by restoring FAD levels (Park et al., 2021). Riboflavin depletion in *C. elegans* promoted adiposity and altered energetic states independent of lifespan genetic dependencies (Yerevanian et al., 2022). In animal models, exposure to environmental toxicants like 6PPD was found to disrupt hepatic riboflavin metabolism, leading to decreased FMN levels and subsequent disturbances in glucose and lipid metabolism (Fang et al., 2024). Similarly, combined exposure to acephate and cadmium in zebrafish (*D. rerio*) induced riboflavin metabolism disorders along with glycolipid metabolism dysfunction (Hu et al., 2023). These findings suggested that disruption of the riboflavin pathway may play a significant role in liver diseases associated with environmental pollutants and HFD.

In this study, six hub targets (IL-6, IL-1β, Casp3, Pparγ, Alb and Tgfβ1) were identified in the FAD and FMN associated liver injury target network. IL-6, IL-1β, and Casp3 are implicated in acute inflammation. A study shows that NIT-1 cells and islets cultured in the presence or absence of 10 microM riboflavin were studied at baseline and after exposure to cytokines (IL-1β), and However, riboflavin prevented the cytokine-induced increase in IL-6 mRNA expression and p38 phosphorylation analyzed by real-time PCR and immunoassay, respectively (Cobianchi et al., 2008). Besides, riboflavin exerted a pronounced anti-inflammatory effect in the polysaccharide-induced inflammation model, both *in vitro*, preventing polysaccharide-induced cell death, and *in vivo*, reducing inflammatory markers (IL-1β, IL-6, and TNF-*α*) and normalizing lung histology (Akasov et al., 2024). Tfgβ1 plays a protective role in renal fibrosis and inflammation (Lan, 2011). A study shows siRNA CCN1 transfection increased the expression of p-NFκB p65, TGF-β1, and p-Smad 2/3 and accelerated the occurrence and development of inflammation and EndMT after hypoxia (Tang et al., 2023). Previous studies have demonstrated that core genes associated with riboflavin include IL-1β, IL-6, and Casp3 (Liu et al., 2025). Furthermore, the Pparγ exhibited an interaction with riboflavin supplementation in affecting BP levels (Holzbach et al., 2025). Furthermore, the Pparγ exhibited an interaction with riboflavin supplementation in affecting BP levels. Research on Alb in relation to riboflavin has not yet been explored, whereas our study has identified Alb as a target. Mechanisms provide important references for future environmental toxicology research related to liver diseases.

## 5 Conclusion

In summary, integrated metagenomic and hepatic metabolomic analyses reveal the pathogenesis of SiNP and HFD co-exposure-induced MAFLD. Gut microbiota dysregulation may act as a key mechanism by mediating riboflavin metabolism, with IL-6, IL-1β, Casp3, Pparγ, Alb and Tgfβ1 serving as critical targets in this metabolic pathway. The current results provide novel mechanistic insights into how environmental nanoparticles like SiNP may interact with dietary factors to promote MAFLD progression through microbiota-metabolite crosstalk, offering potential therapeutic targets for intervention.

## Data Availability

The raw data generated in this study can be found here: https://www.ncbi.nlm.nih.gov, accession PRJNA1455828.

## Footnotes

1
https://www.ebi.ac.uk/chembl/

2
https://ctdbase.org/

3
https://www.genecards.org/

4
http://david.abcc.ncifcrf.gov/
