# Supplementary material for: Combined exposure to silica nanoparticles and high-fat diet modulates metabolism-associated fatty liver disease via the gut–liver axis
Source: Front Microbiol. 2026 May 8;17:1810542. doi: 10.3389/fmicb.2026.1810542 (PMC13194469; doi:10.3389/fmicb.2026.1810542)
Supplement: Supplementary file 1 [file Supplementary_file_1.docx]

**Supplemental Materials**

**Combined exposure to silica nanoparticles and high-fat diet modulates metabolism-associated fatty liver disease via the gut-liver axis**

XueYan Zhang^a1^, Kaifeng Chen^b1^, Yifan Liang^a^, Shuo Sun^a^, Junjie Chen^b^, Qian Wang^b^, Fenghong Wang^c^*, Lei Zhang^a^*

^a^ School of Public Health, Binzhou Medical University, Yantai, Shandong, China

^b^ School of Public Health, North China University of Science and Technology, Tangshan, Hebei, China

^c^ School of Public Health, Inner Mongolia Medical University, Hohhot, Inner Mongolia, China

Table S1. The detailed databases and respective URLs.

Fig. S1. Characterization of SiNP.

Fig. S2. H&E-stained and AB-PAS-stained colon tissue sections reveal structural alterations.

Fig. S3. Visualization of differential microbial abundance.

**Table S1**. The detailed databases and respective URLs

| Database | URL |
| --- | --- |
| NCBI database  HMDB database  METLIN database  ChEMBL database | https:// www.ncbi.nlm.nih.gov  http://www.hmdb.ca/metabolites  http://metlin.scripps.edu/index.php  https://www.ebi.ac.uk/chembl/ |
| CTD database  GeneCards | https://ctdbase.org/  https://www. genecards.org/ |
| DAVID database | http://david.abcc.ncifcrf.gov/ |
| STRING database | https://cn.string-db.org/ |
| Alphafold database | https://alphafold.com |
| Prodigy database | https:// rasca.science.uu.nl/prodigy/ |


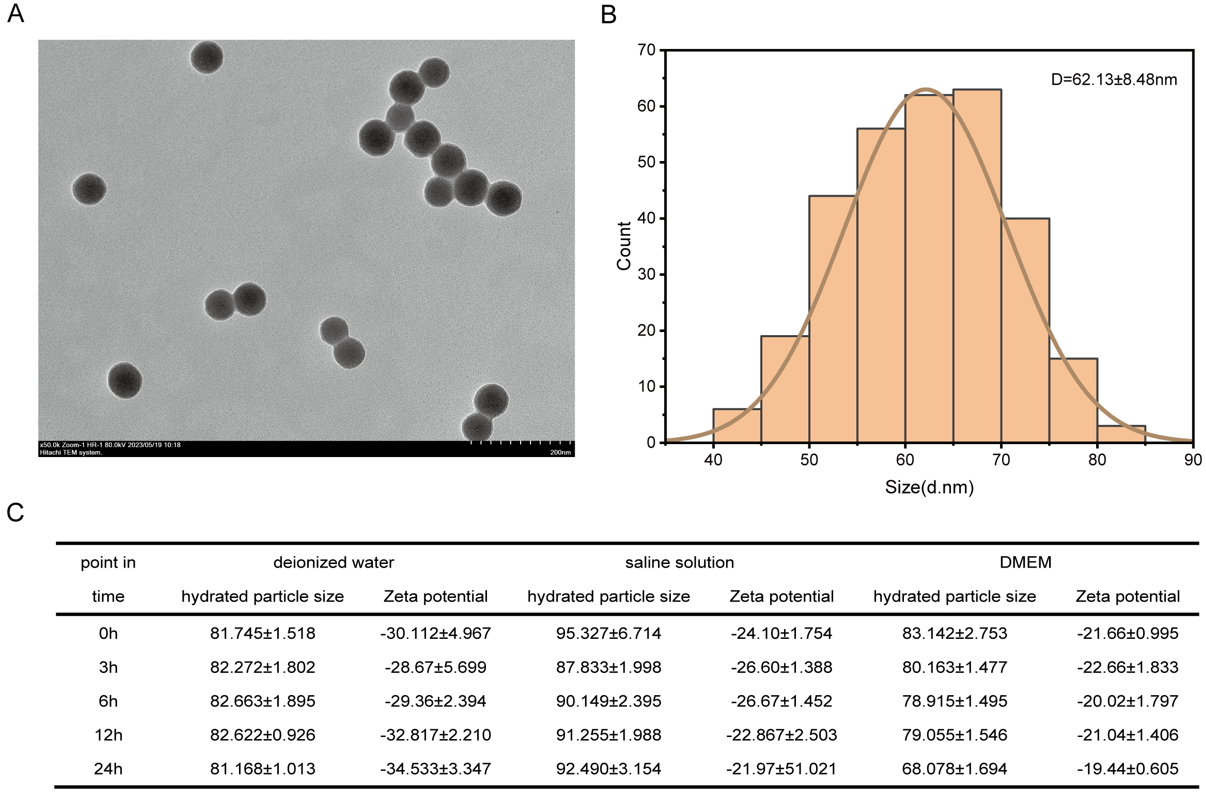


**Fig. S1.** Characterization of SiNP. (A) TEM results-SiNP size (200 nm; 20X); (B) SiNP size distribution; (C) SiNP hydration particle size and zeta potential in different solutions


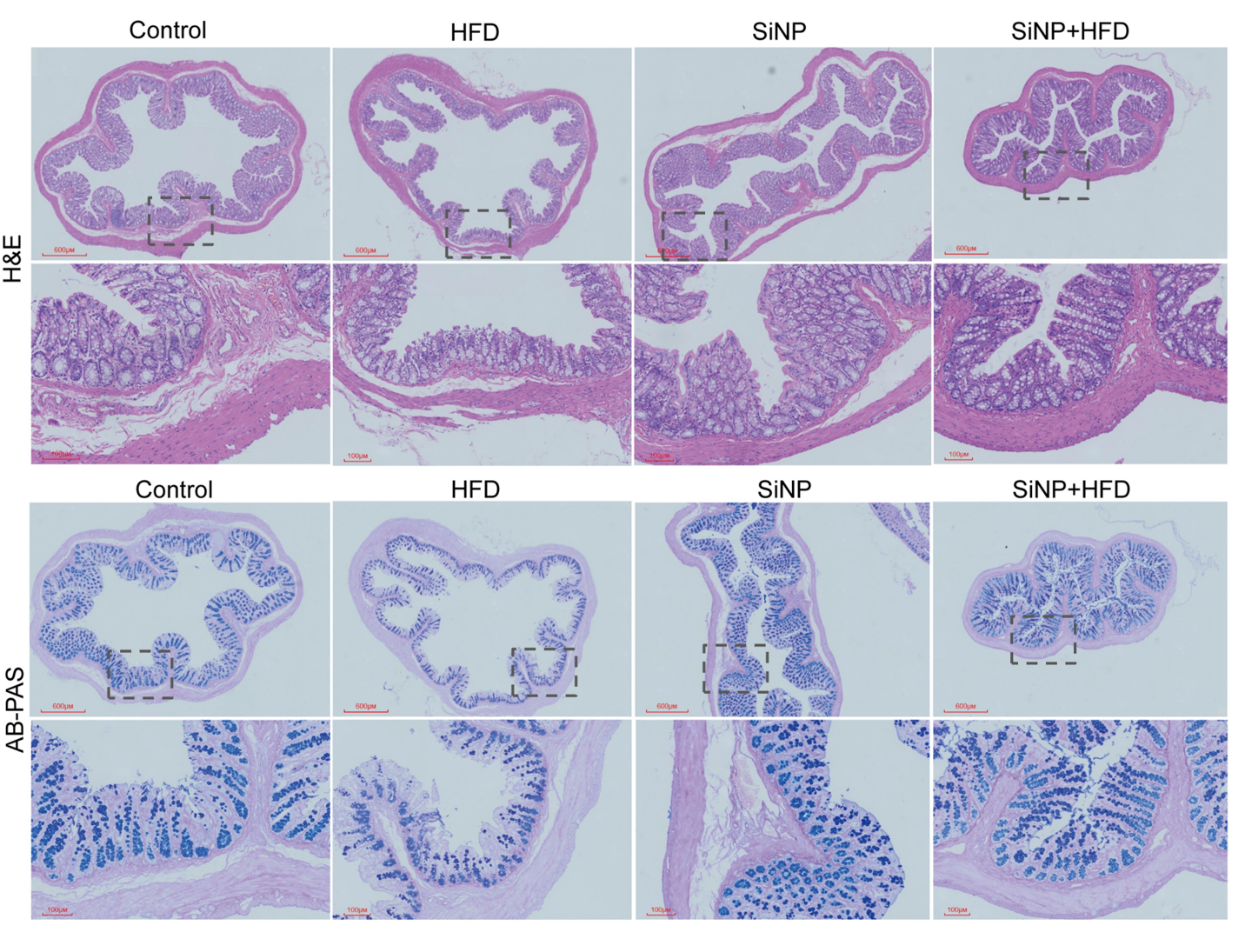


**Fig. S2.** H&E-stained and AB-PAS-stained colon tissue sections reveal structural alterations.


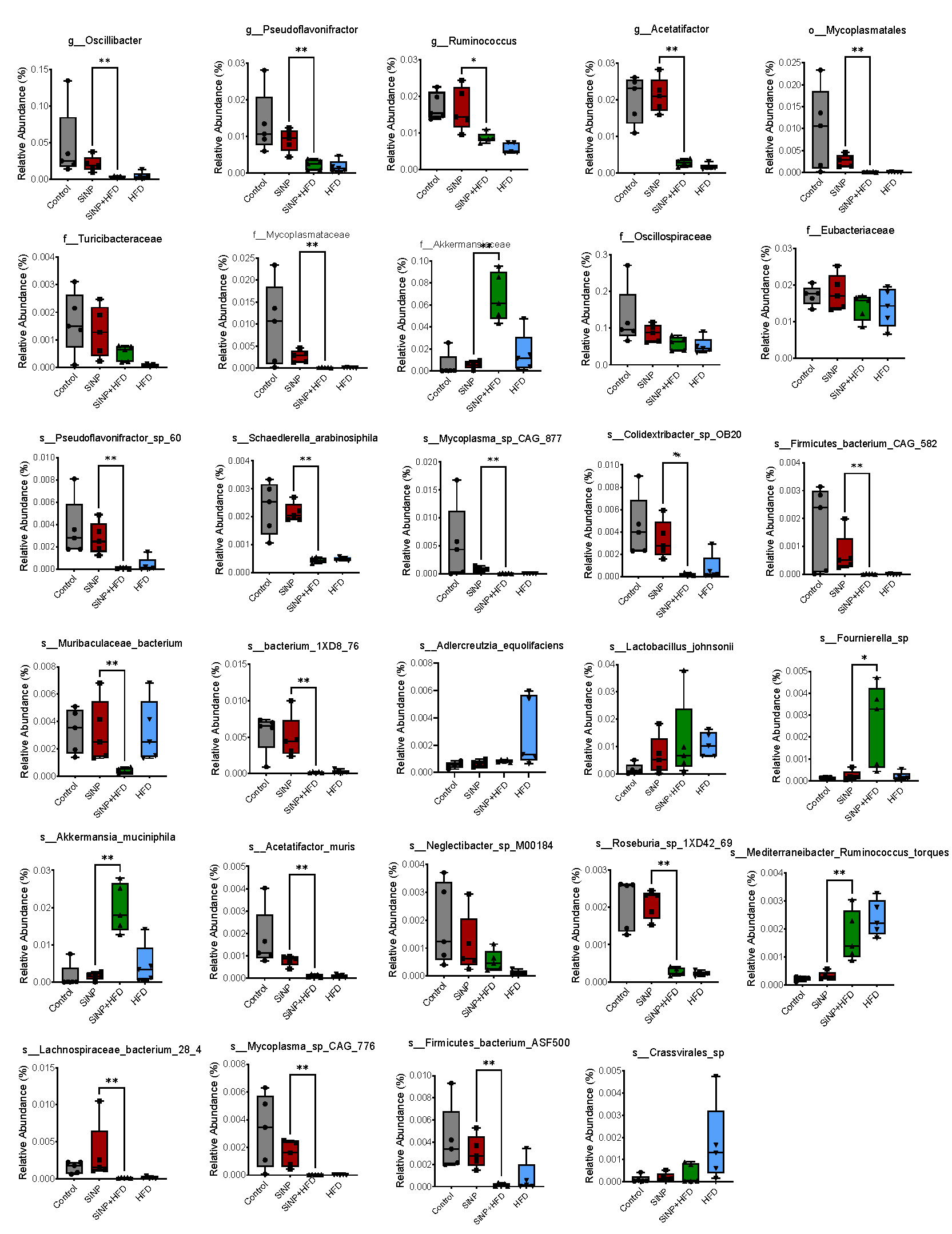


**Fig. S3.** Visualization of differential microbial abundance.
